# The differential effect of maternal dietary patterns on quantiles of Birthweight

**DOI:** 10.1186/s12889-020-09065-x

**Published:** 2020-06-22

**Authors:** Aweke A. Mitku, Temesgen Zewotir, Delia North, Prakash Jeena, Rajen N. Naidoo

**Affiliations:** 1 School of Mathematics, Statistics and Computer Science, College of Agriculture Engineering and Science, University of KwaZulu-Natal, Durban, South Africa; 2 Discipline of Paediatric and Child Health, School of clinical Medicine, College of Health Sciences, University of KwaZulu-Natal, Durban, South Africa; 3 Discipline of Occupational and Environmental Health, School of Nursing and Public Health, College of Health Sciences, University of KwaZulu-Natal, Durban, South Africa; 4 Department of Statistics, Bahir Dar University, Bahir Dar, Ethiopia

**Keywords:** Cohort, Low birthweight, Food frequency questionnaire, Interaction effect, Ordinary regression, Quantile regression

## Abstract

**Background:**

Maternal dietary habits during pregnancy are considered essential for development and growth of the fetus as well as maternal health. It has an effect on the birthweight of infants. However, little is known about the effect of dietary patterns on birthweight in urban South Africa. This study aimed to investigate differential effect of dietary patterns of pregnant women on quantiles of birthweight.

**Methods:**

Data for the study were obtained from a Mother and Child in the Environment birth cohort study in Durban South Africa. Quantile regression was used to investigate the effect of maternal dietary patterns on quantiles of birthweight. Data collection was conducted during the period of 2013 to 2017 in Durban South Africa. Using factor analysis, eight dietary groups were identified from 687 pregnant women in the cohort. Quantile regression analysis was employed to identify the differential effects of the seven dietary groups and demographic factors on the birthweight.

****Result**s:**

The quantile regression estimates at the 50^th^ quantile and the ordinary regression estimates painted the same picture about the conditional mean effect of covariates on the birthweight. But unlike the quantile regression the ordinary regression fails to give insights about the covariates effect disparities at the low and/or upper birthweight quantiles. All the dietary groups show a significant differential effect at different birthweight quantiles. For instance, increased frequency of protein rich foods intake was associated with reduction in birthweight at lower and upper quantiles; increased frequency of junk foods intake has a slight increase in birthweight at the lower quantiles but significantly higher increase at the 95^th^ quantile (*p* < 0.001); increase in consuming vegetable rich foods, reduced birthweight at 95^th^ quantile (p < 0.001). The results further showed that employment (*p* = 0.006) and family size (*p* = 0.002) had differential effects across different birthweight quantiles.

**Conclusions:**

Both maternal undernutrition and overnutrition of protein rich foods, junk foods, snack and energy foods and vegetable rich foods have shown a substantial varying effects on those infants with birthweights in the lower and upper birthweight quantiles.

## Background

Adequate nutrition is vital during pregnancy, both for improved maternal and child health. Women are at risk of having an inadequate nutritional status during pregnancy due to the high nutritional demands during pregnancy. Inadequate maternal nutrition during pregnancy is related to adverse birth outcomes, poor infant survival, respiratory disease in early childhood and then, later in life, cardiovascular diseases and obesity [1–6]. For women living in developing countries, poor quality and quantity of food are major factors for the increased risk of malnutrition during pregnancy [7]. Birthweight has been related to both short and long-term health outcomes and thus, is commonly used as proxy, for studying infant development [8].

Dietary pattern analysis has become a useful tool in epidemiological studies that pursue to better represent a holistic account of the diet [9, 10] and explore the relationship between dietary exposures and health outcomes [11]. It allows the formulation of food-based dietary recommendations [12]. However, individual diets are usually composed of variety of different food items which contain a multitude of nutrients and phytochemicals that function both synergistically and interactively [9]. Dietary patterns characterized by high intakes of vegetables, fruit and dairy products were associated with higher birthweight. Dietary patterns related to low birthweight were often characterized by high loadings of processed and high-fat meat, fats and oils, and sugar rich products in high-income countries [13].

Previous studies showed nutritional status of mothers [14–16] and socio-demographic [16] factors had an association with birthweight. Both very high and very low birthweights are associated with detrimental outcomes. Therefore, modelling risk factors associated with solely lower or upper birthweights using techniques such as linear regression is not necessarily appropriate. Thus, the aim of this study is to assess the effect of maternal dietary pattern on the birthweight quintiles by adjusting the effect of socio-demographic factors.

Most of the statistical techniques used in previous studies for estimating the effect of continuous responses were either with conditional means [17] or through dichotomization, a common practice in epidemiological research. Despite the clinical usefulness contained in the dichotomized outcome, this practice of only considering two outcomes leads to a loss of power and information, due to the choice of the cut-off point of these dichotomous variable [18–20]. Using quantile regression instead of the common methods linear or logistic regression models led to new insights in the data sets [21–24]. In health sciences, quantile regression has become popular in relation to studies of body mass index [23–26]. Accordingly, we adopt quantile regression to model quantiles of birthweight on the nutritional status of mothers [14–16].

## Methods

### Data

The Mother and Child in the Environment (MACE) birth cohort study was conducted in Durban, South Africa. The study had enrolled a cohort of 996 pregnant women at three hospitals in the south (Wentworth Hospital, Prince Mshiyeni Hospital and King Edward VIII Hospital) and at three hospitals in the north (Addington and Mahathma Gandhi, and King George V Hospitals) from March 2013 up to May 2017. The field workers determined if the pregnant women met the inclusion criteria and all pregnant women that did, were invited and recruited into the study. The inclusion criteria were gestational age less than 20 weeks, resident for the full duration of the pregnancy in the geographical area within which the clinic and monitoring station was located, and for the follow-up period of 5–6 years in the cohort. Women with multiple pregnancies as well as 309 miscarriages, loss to follow up and termination of pregnancy were excluded. This study is a retrospective analysis on the remainder 687 enrolled subjects who were followed up during their pregnancy, through to labour and delivery.

Socio-demographic information was taken from enrolment dataset which is conducted with face-to-face interviews by trained enumerators. The enrolment questionnaire also consists variables about antenatal history, place of birth and residential history. For the identification of maternal dietary patterns, a food frequency questionnaire (FFQ), which listed 75 items was administered in the third trimester of their pregnancy. The questionnaire collected information on the 75 food items common to maternal dietary situations during pregnancy. The FFQ specifically designed to reflect South African food consumption habits assessed the use of foods or food groups and the consumption frequency (number of times per day, week or month) as common serving sizes. The selected frequency category for each food item in the FFQ was standardized to times per day. The detailed content of the FFQ and data processing have been described elsewhere [15] and validated [27, 28]. Data was captured at the time of interview using a mobile telephone system, automatically uploading data onto the study database using wireless technology. In South Africa, iron and foliate supplements are standard and all mothers have got these supplements.

To reduce the 75 dietary food items from the FFQ into a set of manageable latent characteristics, with minimal loss of information, exploratory factor analysis of promax orthogonal rotation was performed. The absolute magnitude of the rotated factor loadings greater than 0.30 was used as a threshold value for a variable to belong to a latent group. A scree plot, along with the percentage of variance explained by each factor, resulted eight latent dietary factors for further analyses. Collectively these factors explained 88.33% of the variability within the sample. The summary result with the factor loadings and naming of the latent factors is given in Table 1.

**Table 1:** Factor loadings of different food items in the eight latent dietary factors identified using factor analysis with Promax rotation

Dietary patterns	Food items	Factor loadings coefficient*	Cumulative variance explained (%)
“Snack and energy foods”	Energy bars	0.87486	46.41%
	Energy drinks	0.85251	
	Ice cream	0.76687	
	Chocolate	0.74735	
	Drinking yoghurt	0.70387	
	Milk drinks	0.70378	
	Milkshake	0.66289	
	Fruit salad	0.63208	
	Cheese sauce	0.62704	
	Cheese	0.56129	
	Chicken with skin	0.47309	
	Chips	0.46882	
	Cold meat	0.46529	
	Red meat	0.43206	
	Flame grilled fast food chicken	0.42456	
	Hot dogs	0.40035	
	Sausages	0.39612	
	Fizzy soft drinks	0.38538	
	Fruit juice	0.35977	
	Hamburgers	0.31074	
	Pizza	0.30266	
	Rusks	0.30247	
	Cooking oil	−0.33744	
“Fast foods and spreads”	Dripping	0.76641	11.12%
	Salad dressing low fat	0.76498	
	Fat Holsum	0.74403	
	Schnitzels	0.70762	
	Skimmed	0.6791	
	Chocolate spread	0.60188	
	Bunny chow	0.58556	
	Venison	0.55576	
	Fizzy diet soft drinks	0.51667	
	Fish steamed	0.50548	
	Whole wheat	0.49309	
	Dried fruit	0.47822	
	Red meat fat removed	0.47779	
	Cookies	0.45296	
	Fried fast food chicken	0.4281	
	Pasta	0.42541	
	Organ meat	0.40937	
	Nuts and peanuts	0.36348	
	Pizza	0.34863	
	Pies and sausage rolls	0.34244	
	Vetkoek	0.33541	
	Rusks	0.32428	
	Hot dogs	0.31508	
	Butter	0.3194	
	Fizzy soft drinks	−0.31292	
“Junk foods”	Butter	0.62300	10.45%
	Sweets	0.60625	
	Muffins	0.59219	
	Chips	0.56772	
	Mixed salad	0.56106	
	Fruit juice	0.5389	
	Fresh fruit	0.53471	
	Fizzy soft drinks	0.42281	
	Vetkoek	0.39649	
	Coffee creamer	0.38703	
	Cooking oil	0.38482	
	Hamburgers	0.36097	
	Cooked vegetables	0.35781	
	Cereals Rice Crispies	0.31854	
	Soft margarine	−0.77394	
“Protein rich foods”	Fried fish	0.67237	6.35%
	Fish tinned	0.54825	
	Fried fish in fat	0.52289	
	Eggs cooked or poached	0.46767	
	White brown bread	0.43925	
	Potato chips	0.4079	
	Chicken with skin	0.40252	
	Chicken without skin	−0.34525	
	Red meat fat removed	−0.40646	
“Starch foods”	Potato	0.66418	4.56%
	Breakfast cereals	0.52251	
	Potato with fat	0.47055	
	Legumes	0.45527	
	Full cream	0.40847	
	Cooked vegetables	0.36178	
	Cheese sauce	0.35377	
	Pasta	0.32992	
	Jam	0.32221	
“Nuts and rice foods”	Peanut butter	0.55276	3.63%
	Nuts and peanuts	0.39124	
	Rice mealie rice	0.34955	
“Vegetable rich foods”	Vegetables	0.61485	3.13%
	Organ meat	0.40243	
	Butter	−0.34338	
“Alcoholic drinks”	Shooters	0.80795	2.67%
	Cocktails	0.79207	

*Factor loadings ≥3.0 or ≤ −3.0. Food groups are sorted by size of loading coefficients

### Statistical analysis

We explored the data on quantiles of birthweight and observed that extreme birthweight outcomes occur over several maternal strata; including marital status, educational level, employment status and annual income, as well as infant strata, specifically gender. The quantile regression model provides the effects of maternal diet across the distribution of birthweight taking into consideration outliers. In other words, the quantile e regression is particularly useful with data that are heterogeneous in that the tails and the central location of the conditional distributions vary differently with the covariates [22]. The effect of covariates on quantiles of the response distribution are pertinent. The covariates considered in this study were the eight latent dietary factors and socio-demographic factors. Quantile regression for a set of covariates, **X**, on the (***τ ×*100**)^***th***^ quantiles of **y** is given by

$$ {\boldsymbol{Q}}_{\boldsymbol{\tau}}\;\left(\boldsymbol{y}/\boldsymbol{X}\right)={\boldsymbol{X}}^{\boldsymbol{t}}\;\boldsymbol{\beta}\;\left(\boldsymbol{\tau} \right)+\boldsymbol{\varepsilon} $$

where 0 < *τ* < 1 and *ε* = (*ε*_1_, …, *ε*_*n*_)^*t*^ is a vector of independent errors. The parameter estimates, ***β***(***τ***) have the same interpretation as those of any other linear model, i.e. each *β*_*j*_(*τ*) coefficient can be interpreted as the marginal change in the (***τ ×*100**)^***th***^ quantile, due to the marginal change in corresponding j^th^ covariate [22, 29, 30]. The quantile regression coefficients are computed by minimizing the asymmetric weighted sum of absolute errors through linear programing methods:

$$ \underset{\boldsymbol{\beta}}{\mathbf{\min}}\left[\sum \limits_{\boldsymbol{i}:{\boldsymbol{\beta}}_{\boldsymbol{i}}\mathbf{\ge}{\boldsymbol{x}}^{\prime}\boldsymbol{\beta}}\boldsymbol{\tau} \left|{\boldsymbol{\beta}}_{\boldsymbol{i}}-{\boldsymbol{x}}_{\boldsymbol{i}}^{\prime }{\boldsymbol{\beta}}^{\boldsymbol{\tau}}\right|+\sum \limits_{\boldsymbol{i}:{\boldsymbol{\beta}}_{\boldsymbol{i}}\mathbf{\le}{\boldsymbol{x}}^{\prime}\boldsymbol{\beta}}\left(\mathbf{1}-\boldsymbol{\tau} \right)\left|{\boldsymbol{\beta}}_{\boldsymbol{i}}-{\boldsymbol{x}}_{\boldsymbol{i}}^{\prime }{\boldsymbol{\beta}}^{\boldsymbol{\tau}}\right|\right] $$

The model was built by fitting all the main effects followed by sequential assessment of whether any interaction terms need to be incorporated in to the model. Consequently, only two two-way interaction term of employment status with marital status and maternal education improved the main effect quantile regression model fit. Outliers can adversely influence the fit of the model thereby invalidating the appropriate statistical inferences [31]. However, quantile regression is fairly robust to outliers as their influence functions are bounded in the Y-space [22]. Existence of single case outlier diagnostic can be checked based on the standardized median absolute deviation of residuals [32]. The robust and multivariate location and scale diagnostics computed using the minimum covariance determinant (MCD) method were applied to expose all the single case high leverage points and outliers [33]. We used the standardized residuals and Quantile-Quantile plots for checking the goodness of fit of the model. All the statistical analyses were performed using SAS, version 9.4.

## Results

The overall mean birthweight of the 687 children in the birth cohort was 3107.0 g (g) with a median of 3140.0 g. The data included 357 male and 328 female infants, with 80.6% of mothers being single and 79.5% with high school education. Majority of the women in the cohort were unemployed (81.5%), have no personal income (47.9%) and 48.8% of them were nulliparous. The 95^th^ quantile of birthweight was higher in infants born to women who were married, primary or less education or were primaparous. Male offspring, older maternal age, lower education were observed to have lower birthweight at 5^th^ quantile. The quantiles of birthweight by socio-demographic characteristics are summarized in Table 2.

**Table 2:** Descriptive statistics and quantiles of birthweight by socio-demographic characteristics of women in MACE birth cohort

Variable	N (%)	P_5_	P_10_	P_25_(Q_1_)	Median	P_75_(Q_3_)	P_90_	P_95_
Marital status								
Married	93 (13.6)	2084	2400	2900	3300	3560	3990	4031
Living together	39 (5.7)	2080	2400	2800	3112	3430	3800	3970
Single	554 (80.8)	2090	2400	2840	3115	3400	3720	3980
Maternal age								
Teenage (less than 25 yrs)	116 (16.9)	2070	2331	2740	3060	3300	3560	3640
Prime of fertility (25–29 yrs)	416 (60.6)	2100	2440	2876	3160	3430	3800	4000
Adult age (30 years and above)	155 (22.6)	2031	2340	2800	3170	3400	3800	4000
Maternal education								
Primary or less	16 (2.3)	1570	1800	2775	3210	3495	3870	4260
Secondary school	546 (79.5)	2083	2400	2815	3105	3400	3710	3920
College or university	125 (18.2)	2096	2410	2960	3210	3480	3970	4120
Employment								
Employed	127 (18.5)	2080	2360	2820	3100	3480	3850	4010
Unemployed	560 (81.5)	2090	2400	2860	3140	3400	3730	4000
Maternal annual income								
No personal income	329 (47.9)	2090	2400	2885	3160	3400	3800	4000
Less than R30000	276 (40.2)	2090	2400	2800	3100	3400	3700	3980
R30000 and above	82 (11.9)	2083	2530	2840	3150	3480	3800	3970
Parity								
Nulliparous	334 (48.8)	2080	2360	2790	3100	3340	3630	3850
Primaparous	220 (32.1)	2079	2060	2870	3230	3500	3895	4045.5
Multiparous	131 (19.1)	2280	2500	2900	3150	3410	3800	3990
Infant Gender								
Male	359 (52.3)	2084	2370	2860	3170	3450	3900	4060
Female	328 (47.7)	2090	2410	2830	3125	3370	3600	3810

**Fig. 1:**
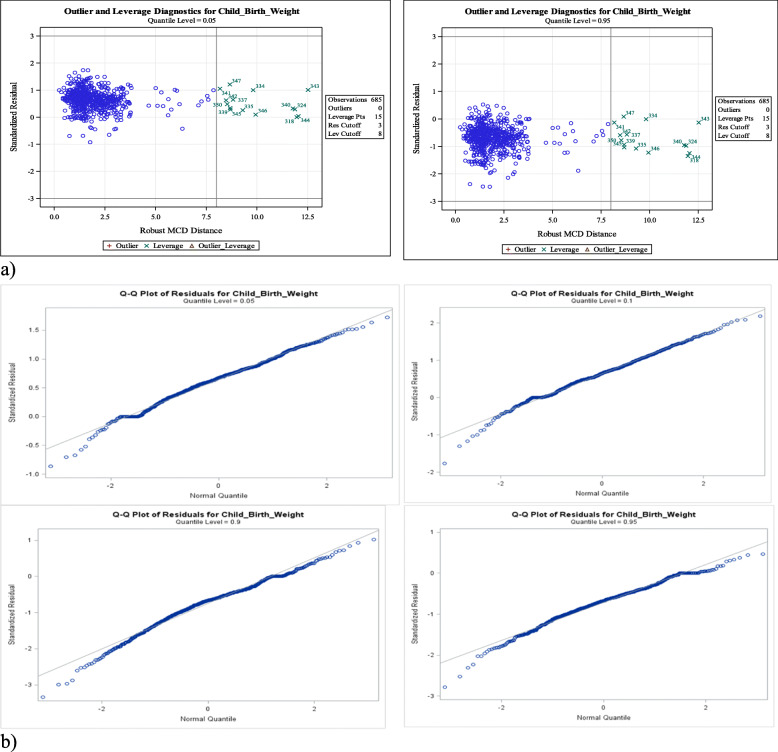
Diagnostic plots for the final quantile regression model

Table 3 shows the results from estimation of the final quantile regression model and ordinary least squares regression. Before we make any inference from the model results we examine its goodness of fit. All the goodness of fit assessment results in Fig. 1 showed that the final model fits the data adequately.

**Table 3:** Quantile regression parameter estimates and 95% confidence intervals of dietary latent factors for the 5^th^, 10^th^, 25^th^, 50^th^, 75^th^, 90^th^, and 95^th^ quantiles of birthweight, adjusted for demographic and socio-economic characteristics

Quantiles	5^th^	10^th^	25^th^	50^th^	75^th^	90^th^	95^th^	OLS
	**(95% CI)**	**(95% CI)**	**(95% CI)**	**(95% CI)**	**(95% CI)**	**(95% CI)**	**(95% CI)**	**(95% CI)**
Intercept	2741.8^**^ (2570.2, 2913.4)	3060.9^**^ (2612.9, 3508.8)	3067.5 (2785.1, 3349.9)	3190.4** (3019.4, 3361.4)	3514.7** (3313.5, 3715.8)	4095.5** (3871.7, 4319.3)	4756.6** (4592.7, 4920.5)	3395.1**(3173.6, 3616.7)
Snack and energy protein foods factor	4.6 (38.8, 47.9)	96.3 (−16.8, 209.5)	22.6 (−48.8, 93.9)	−0.4 (−43.6, 42.7)	2.5 (−48.3, 53.3)	52.6 (−3.9, 109.1)	65.8** (24.4, 107.2)	17 (−38.7, 73.2)
Fast foods and spreads factor	2.2 (−37.0, 41.4)	−50.9 (− 153.4, 51.4)	−2.0 (−66.5, 62.6)	− 15.2 (− 54.3, 23.9)	36.6 (−9.4, 82.6)	−11.4 (−62.5, 37.8)	−26.9 (−64.4, 10.5)	−11.5 (−62.1, 39.2)
Junk foods factor	27.6 (−18.6, 73.8)	21.8 (− 99.2, 141.8)	32.6 (− 43.4, 108.6)	34.0 (− 12.0, 80.1)	25.4 (− 28.7, 79.5)	−5.8 (− 66.0, 54.4)	81.5** (37.4, 125.6)	41.8 (− 17.8, 101.4)
Protein rich foods factor	− 62.2** (− 102.8, − 21.5)	−76.7 (− 182.9, 29.4)	−71.0 (− 137.9, − 4.1)	−16.3 (− 56.8, 24.2)	14.0 (− 33.6, 61.7)	3.4 (− 49.6, 56.5)	− 64.9** (−103.8, − 26.1)	−20.3 (−72.8, 32.2)
Starch foods factor	42.5* (5.4, 79.5)	15.5 (−81.2112.2)	−29.5 (− 90.5, 31.5)	10.2 (− 26.7, 47.2)	25.3 (− 18.1, 68.7)	39.9 (− 8.4, 88.3)	−8.2 (− 43.6, 27.2)	10.7 (− 37.2, 58.5)
Nuts and rice foods factor	46.7* (6.6, 86.8)	94.2 (− 10.5, 194.9)	37.0 (− 29.0, 103.0)	47.2* (7.2, 87.1)	41.1 (−5.8, 88.1)	14.6 (− 37.7, 66.9)	76.4** (38.4, 114.9)	42.1 (−9.7, 93.8)
Vegetable rich foods factor	119.5** (74.4, 164.6)	77.2 (− 40.5, 194.9)	38.0 (− 36.3, 112.2)	14.4 (− 30.5, 59.4)	5.7 (− 47.2, 58.5)	1.9 (− 56.9, 60.8)	−75.7** (− 118.8, − 32.7)	5.6 (− 52.6, 63.8)
Alcoholic drinks factor	−46.6 (− 194.8, 101.6)	−140.9 (− 527.9, 245.9)	− 288.2 (− 532.2, − 44.3)	−84.8 (− 232.6, 62.9)	− 149.9 (− 323.6, 23.8)	− 426.6 (− 619.9, − 233.3)	− 558.1* (− 699.6, − 416.6)	− 195.9* (− 387.3, −4.6)
Marital status (REF=Single)								
Married	−82.7 (189.1, 23.8)	286.4 (−143.4, 716.2)	37.5 (−137.7, 212.7)	−207.6** (101.5, 313.7)	194.5** (69.7, 319.3)	174.0* (35.2, 32.8)	36.3 (−65.4, 137.9)	115.7 (− 21.8, 253.0)
Living together	283.0** (118.4, 447.7)	84..5 (− 193.3, 362.4)	54.3 (− 216.7, 325.3)	− 71.8(− 235.9, 92.3)	−16.4 (− 209.4, 176.6)	−185.2 (− 399.9, 29.6)	−309.4** (− 466.7, − 152.2)	−13.3 (− 225.8, 199.3)
Maternal age (REF=Prime of fertility)								
Teenage	−1.4 (−95.0, 92.2)	14.2 (− 230.2, 258.5)	− 41.9 (− 195.9, 112.2)	−27.0 (− 120.3, 66.3)	−51.7 (− 161.4, 58.0)	−153.4* (− 275.5, − 31.3)	− 201.2** (− 290.6, − 111.8)	− 40.2 (− 161.0, 80.7)
Adult age(30 years and above)	− 167.5** (− 255.4, − 79.5)	213.2 (− 442.7, 16.3)	−106.9 (− 251.6, 37.9)	−53.8 (− 141.4, 33.9)	−121.1* (− 224.1, − 17.9)	− 167.3** (− 282.0, − 52.6)	− 221.8** (− 305.8, − 137.8)	−116.7* (− 230.2, − 3.1)
Maternal education (REF = College or University)								
Primary or less	− 435.8* (− 673.3, − 198.2)	− 666.2* (− 1286.3, − 46.1)	− 437.1* (− 828.1, − 46.1)	−53.7 (− 290.4, 183.1)	−6.9 (− 285.4, 271.5)	−242.8 (− 552.7, 67.0)	−105.4 (− 332.2, 121.4)	− 250.9 (− 557.5, 55.8)
High school	− 90.6 (− 185.9, 4.7)	−281.2* (− 529.8, − 32.6)	−112.5 (− 269.3, 44.2)	−51.1 (− 146.0, 43.8)	−58.8 (170.4, 52.9)	−160.2* (− 284.4, − 35.9)	− 215.5** (− 306.5, − 124.6)	−135.9* (− 258.9, − 12.9)
Employment (REF=Unemployed)								
Employed	−188.4 (− 387.9, 11.3)	− 569.5* (− 1090.5, − 48.4)	−95.7 (− 424.2, 232.9)	−78.0 (− 120.9, 276.9)	86.3 (− 147.7, 320.3)	−21.3 (− 281.6, 239.1)	− 174.1 (− 364.8, 16.5)	−57.2 (− 314.9, 200.5)
Maternal annual income (REF = R30000 and above)								
No personal income	− 108.3 (− 219.2, 2.6)	− 80.2 (− 369.6, 209.2)	76.4 (− 106.1, 258.9)	31.9 (−78.6, 142.4)	82.6 (− 47.4, 212.6)	88.4 (− 56.2, 233.0)	21.2 (− 84.7, 127.1)	39.0 (− 104.1, 182.1)
Less than R30000	− 153.7* (− 271.3, − 36.1)	− 157.1 (− 464.1, 149.8)	− 62.9 (− 256.5, 130.6)	−23.4 (− 140.6, 93.8)	28.9 (− 108.9, 166.7)	40.1 (− 113..3, 193.5)	− 192.4** (− 304.7, − 80.1)	−61.5 (− 213.3, 90.3)
Infant Gender (REF = Male)								
Female	81.2* (18.2, 144.3)	68.1 (− 96.3, 232.6)	58.2 (− 45.5, 161.9)	− 34.4 (− 97.2, 28.4)	− 106.7** (− 180.6, − 32.9)	− 187.6** (− 269.8, − 105.5)	−219.4** (− 279.5, − 159.2)	−67.8 (− 149.2, 13.5)
Parity (REF = Multiparous)								
Nulliparous	− 246.4** (− 348.5, − 144.3)	−300.1* (− 566.6, − 33.6)	−182.9 (− 350.9, − 14.9)	− 91.1 (− 192.8, 10.7)	−119.6 (− 239.2, 0.1)	− 229.3** (− 362.5, − 96.2)	− 327.3** (− 424.7, − 229.8)	−160.1* (− 291.9, − 28.4)
Primaparous	− 179.1** (− 274.6, − 83.6)	−228.8 (− 478.1, 20.6)	−130.4 (− 80.1, − 287.6)	25.6 (− 69.6120.8)	58.8 (− 53.1, 170.8)	−47.1 (− 171.6, 77.5)	− 107.4* (− 198.6, − 16.2)	−10.5 (− 133.8, 112.8)
Family Size	−73.6** (− 93.4, − 51.9)	−19.8 (− 74.1, 34.4)	−5.5 (− 39.7, 28.6)	15.8 (−4.9, 36.5)	−3.9 (− 28.2, 20.5)	−9.4 (− 36.5, 17.7)	−51.3** (− 71.1, − 31.5)	−12.6 (− 39.4, 14.2)
Married* employed	314.0** (80.5, 547.5)	97.5 (− 512.0, 706.9)	12.0 (− 372.3, 396.3)	−223.3 (− 456.0, 9.4)	− 190.2 (− 463.8, 83.5)	− 277.5 (− 581.9, 27.1)	− 429.0** (− 651.9, − 206.1)	−65.9 (− 367.4, 235.4)
Living together* Employed	− 172.8 (− 474.1, 128.5)	−318.6 (− 1104.9, 467.8)	−110.6 (− 606.4, 385.2)	288.2 (−12.0, 588.0)	295.6 (− 57.4, 648.7)	218.2 (− 174.7, 611.1)	361.6* (73.9, 649.2)	127.2 (− 261.7, 516.0)
Single*Unemployed (REF)								
Primary or less* Employed	1392.0** (742.8, 2041.2)	1666.0 (28.6, 3360.7)	753.8 (− 314.7, 1822.4)	382.8 (− 264.3, 1029.8)	664.3 (− 96.7, 1425.2)	364.9 (− 481.9, 1211.6)	98.1 (− 521.8, 718.1)	779.1 (− 58.9, 1617.2)
High school * Employed	237.6* (30.8, 444.5)	634.8* (94.9, 1174.7)	50.5 (−289.9, 391.0)	−137.5 (−343.6, 68.6)	−69.3 (− 311.7, 173.1)	98.9 (− 170.9, 368.6)	296.4** (98.9, 493.8)	76.3 (− 190.6, 343.3)
College or University*Unemployed (REF)								

^**^Significant at 0.01 level^*^significant at 0.05 level

From Table 3, the confidence intervals of the ordinary regression and the quantile regression at the 50^th^ quantile have a considerable overlap. Otherwise quantile regression estimates confidence intervals lie outside the confidence intervals for the ordinary least squares regression, suggesting that the effects of these covariates may not be constant across the conditional distribution quantiles. And hence justifies the importance of quantile regression to have a better picture in the whole spectrum of the birthweight quantile.

**Fig. 2:**
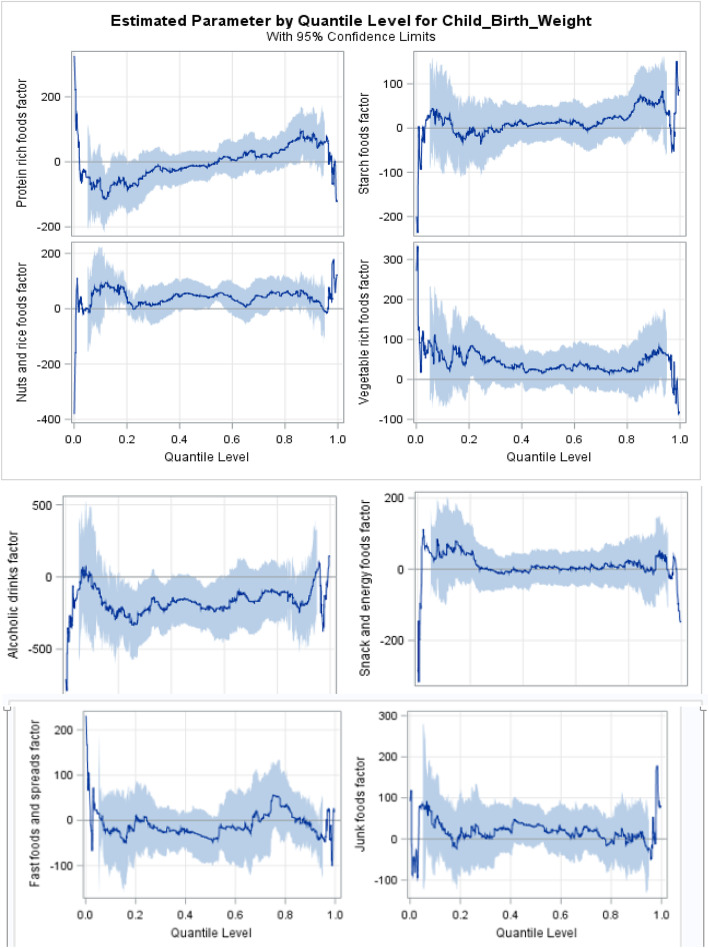
Differential effect of dietary patterns across quantiles of birthweight in the cohort of pregnant women

In order to avoid redundancy in the interpretation of the results in Table 3 and Fig. 2, we interpret few. Vegetable rich foods consumption during pregnancy increased birthweight at lower quantiles. This increase was significant at the 5^th^ quantile (*p* = 0.001). However, in the 95^th^ quantile, increase in consumption of vegetable rich foods had resulted in birthweight reduction. An increased frequency of junk foods intake by mothers was also associated with a slight increase in birthweight at the lower quantiles and significantly higher increase at the 95^th^ quantile (*p* < 0.001). The results also indicated that consumption of snack and energy foods (p = 0.001), nuts and rice foods (*p* < 0.001) and junk foods (p < 0.001) during pregnancy increased the infant birthweight at the 95^th^ quantiles of birthweight. Similarly, higher frequency of consuming nuts and rice foods by mothers is associated with increased birthweight in the 50^th^ quantile (*p* = 0.021).

Mothers who consume protein rich foods with a higher frequency, tend to give birth to infants with significantly lower birthweight, as evidenced in the 5^th^ and 95^th^ quantile (*p* < 0.001). However, an increased frequency of protein rich foods intake increased birthweight of an infant at the75^th^ and 90^th^ quantiles. Female infants had a lower birthweight at the upper quantiles (*p* < 0.001) than males.

**Fig. 3:**
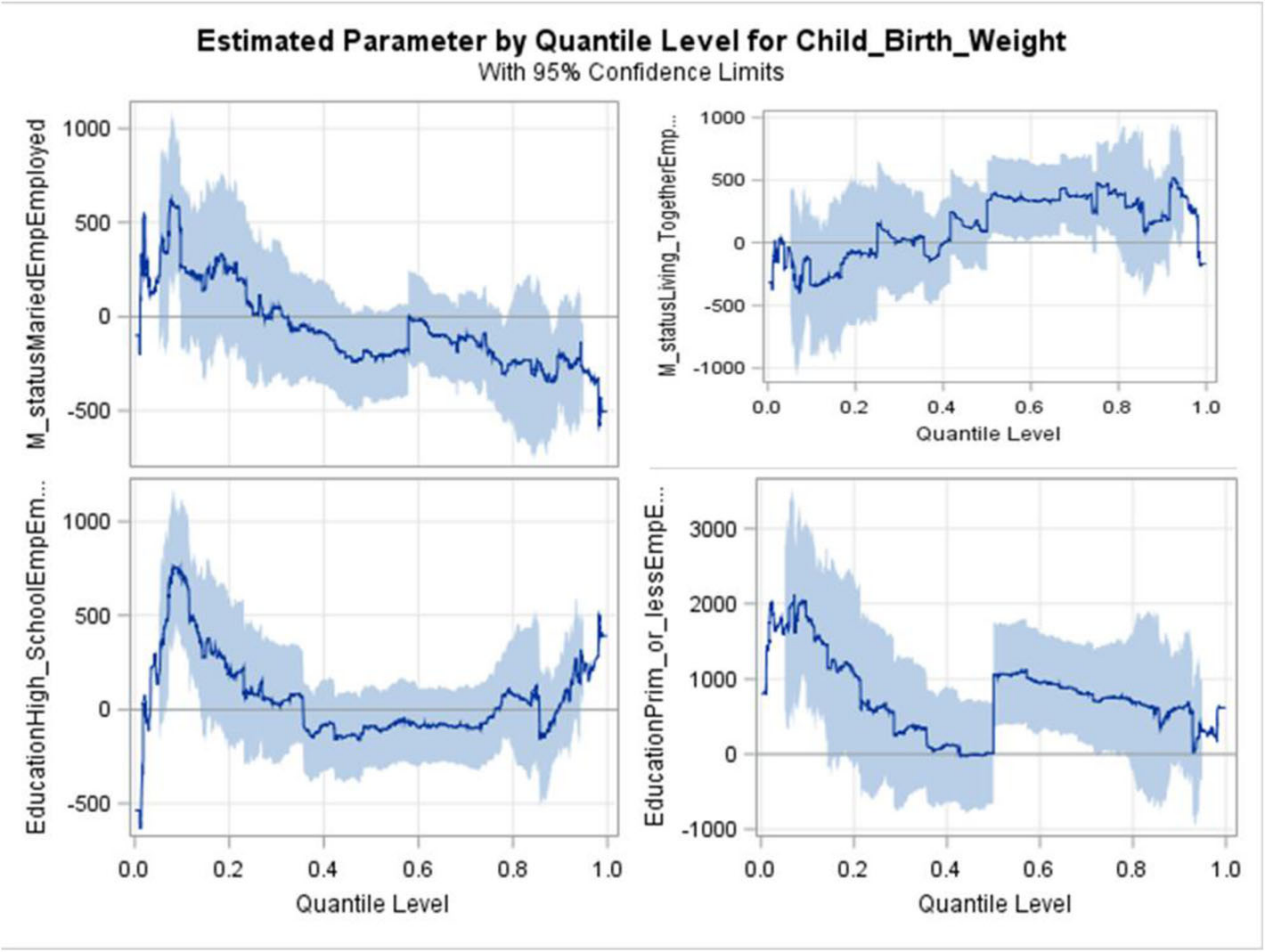
Association between interaction estimates of employment status with marital status and education of pregnant women across different quantiles of birthweight

The two-way significant interactions were maternal employment status with marital status and maternal employment status with maternal education (Fig. 3). Infants born to employed women with marital status of living together weighed less than infants born to married mothers in the lower tail of the birthweight distribution but are more likely to have high birthweight at the upper tail quantiles. The interaction between maternal employment and education had a large positive effect on birth weight, especially in the lower tail; this difference is smaller in the upper quantiles of the distribution. For instance, at the 5^th^ and 10^th^ quantiles, infants from employed mothers with some primary school or less education, had 1670 g (*p* < 0.001) and 1824 g (p < 0.001) higher birthweights respectively than those from unemployed mothers with college or university education.

## Discussion

This study showed, through the use of novel statistical approach, that protein rich foods dietary pattern had significant differential effects in the lower and upper quantiles of birthweight in Durban, South Africa. Our findings further suggest that vegetable rich foods and starch foods dietary patterns showed a protective effect at the 5^th^ quantile of birthweight. Quantile regression models allowed for exploration of differential effects of dietary patterns across quantiles of birthweight adjusting for important demographic and socio-economic factors in MACE birth cohort. The differential effect of dietary patterns on quantiles of birthweight has not been previously described.

Previous studies that examined the association between maternal dietary patterns during pregnancy and birth outcomes, particularly in the presence of a high burden of low birthweights in the data set, used ordinary least square and logistic regression [11, 15, 17, 34]. The quantile regression insight on differential effect of maternal dietary on the different quantiles of birthweight.

Consumption of a traditional dietary pattern (potatoes, meat, vegetables) in early pregnancy, has been found in other studies to reduce the risk of having a low birthweight infant [11, 17]. Our study found further evidence in support of these studies, with consumption of vegetable rich foods and starch foods having a protective effect at the 5^th^ quantile of birthweight. Unlike to these studies, our findings found that consumption of vegetable rich foods had associated with birthweight reduction at the 95^th^ quantile. However, this is consistent with a study among British women that found processed and vegetarian diets were associated with the lowest birthweight [35]. A study in Brazil demonstrated that snack dietary patterns of mothers was associated with increase in birthweight [34]. In line with this, our findings showed increased intake of snack and energy foods by mothers during their pregnancy was associated with increased birthweight at lower and upper tail of birthweight. A review on studies from high-income countries found junk foods dietary pattern is related to low birthweight [13]. On the contrary, the findings of our study indicated that maternal consumption of junk foods during pregnancy was associated with increased offspring birthweight at the 95^th^ quantile. In line with our study, junk food diet characterized by high consumption of fast foods, soft drink, processed meat, or chips frequently during pregnancy was associated increased risk of having a baby with a high birthweight in Australia [36].

In the 5^th^ quantile, our study revealed that consumption of protein rich foods by mothers is associated with decreased birthweight. This is similar to what was found in studies in Ghana and Denmark, which indicated that red and processed meat diets during pregnancy, was associated with an increased risk of infants with lower birthweights [15, 37]. It is also noted that in a low-income cohort of US women, high protein intake was associated with reduced birth weight [38]. Moreover, the quantile regression modeling in our study showed evidence of a varied effect of protein rich foods consumed by mothers, throughout the conditional distribution of birthweight. For instance, women with more frequent consumption of protein rich foods, were associated with giving birth to infants with increased birthweight at the 75^th^ and 90^th^ quantile and these association was with a reduced birthweight at the 95^th^ quantile.

Completing at least upper secondary education was found protective against low birthweight infants [39, 40]. Adverse socio-economic conditions such as maternal unemployment [40] and education level [41] have been linked to low birthweight in other studies. Unlike these studies, our study found an interaction effect of maternal employment with education and marital status. i.e. unemployed women with college or university education were more likely to have a low birthweight infant. The findings of the present study indicated that infants from employed and unmarried women, tend to have lower birthweight in the bottom lower (5^th^) quantile, but more likely to be macrosomia in the top upper tail (95^th^) quantile. This may be attributed to the job loadings on women during pregnancy. The quantile regression in this study showed that employment and family size had a differential effect across the different quantiles of birthweight. In higher quantiles of birthweight, female infants had consistently lower birthweight. This results corroborate what other authors who used linear regression [42] and logistic regression [43] found, i.e. male infants were more likely to be heavier at birth, compared with female infants.

An important strength of our study is that we were able to obtain detailed dietary data from an ongoing birth cohort, and using this rich dataset, we were able to describe, through factor analysis, a typical dietary pattern in a developing country, low socio-economic community utilizing both traditional and “western” dietary patterns. The additional strength of this study is that use of novel statistical approach, quantile regression, which is a very useful tool for data that are heterogeneous, in the sense that the tails and the central location of the conditional distributions vary differently with the levels of covariates and it is also robust, as it makes no distributional assumption about the error term in the model. A limitation of the study was the varying description of similar food types among participants (eg. the frequent use of trade names or local terms in describing intake), which may have resulted in high loading at different factors.

## Conclusions

Food frequency questionnaires (FFQ) have been used in the study of dietary patterns among pregnant females. The advantage of this approach is that it identifies local dietary intake. In order to minimize the recall bias, the dietary data was collected in the third trimester of pregnancy, not at neonatal. Exploratory factor analysis data reduction was employed to transform the large set of correlated variables into smaller sets of non-correlated variables, known as factors, allowing a better understanding of the dimensions underlying the initial variables. Quantile regression allowed modeling the differential effect of maternal dietary patterns adjusting for socio-demographics on the entire quantile of birthweight spectrum. This would have been missed if traditional regression methods had been employed as it models at the average birthweight. The quantile regression model identified substantially differential effects of protein rich foods, parity and infant gender in the lower and upper distribution of birthweight. Moreover, vegetable rich foods and starch foods dietary patterns showed a protective effect at the 5^th^ quantile of birthweight. Future studies need to consider other indicators such as gestational age, birth length and head circumference as measures of birth outcomes to better explore the effects of maternal dietary patterns.

## Data Availability

The data that support the findings of this study are available from MACE study but restrictions apply to the availability of these data, which were used under license for the current study, and so are not publicly available. Data are however available from the authors upon reasonable request and with permission from MACE study.

## Abbreviations

FFQ: Food Frequency Questionnaires
MACE: Mother and Child in the Environment
MCD: Minimum Covariance Determinant
